# Intergenerational trauma and war-induced PTSD in Kosovo: insights from the Albanian ethnic group

**DOI:** 10.3389/fpsyg.2023.1195649

**Published:** 2023-08-10

**Authors:** Zamira Hyseni Duraku, Genta Jahiu, Donjeta Geci

**Affiliations:** Department of Psychology, University of Prishtina “Hasan Prishtina”, Prishtina, Kosovo

**Keywords:** post-traumatic stress disorder, intergeneration trauma, traumatic experiences, youth, parents, social support

## Abstract

**Introduction:**

War has profound and deep-rooted ramifications for individuals and societies. War-induced post-traumatic stress disorder (PTSD) is highly prevalent in Kosovo. This study aimed to obtain insights into the prevalence of perceived PTSD symptoms and their relation to the traumatic experiences of two generations: parents (survivors of the Kosovo War) and youth (children born after the Kosovo War), with an emphasis on the Albanian ethnic group. These experiences were then compared to understand intergenerational trauma. The study also aimed to identify the factors affecting PTSD prevalence, the role of social support, and the participants’ experience with mental health services.

**Method:**

A total of 237 Kosovar Albanians (121 parents, 116 youth) from all seven districts of Kosovo were included in this study. Study variables were measured using the PTSD Checklist, the Life Events Checklist, Criterion A, and the Multidimensional Scale of Perceived Social Support.

**Results:**

The results revealed that the youth had significantly higher levels of perceived PTSD symptoms and lower levels of perceived support than their parents. Youth whose parents had PTSD were more prone to experiencing PTSD symptoms than those whose parents did not have PTSD. These youth also experienced significantly more traumatic situations, such as exposure to sudden violent death or accidental death, assault with a weapon, sexual assault, and captivity. Participants with perceived PTSD and lower perceived social support needed mental health interventions significantly more than those without PTSD symptoms.

**Discussion:**

The findings emphasize the importance of addressing the intergenerational nature of PTSD and identifying factors affecting its prevalence, including social support and access to mental health services. The study underscores the need for a comprehensive approach to examine the complex and diverse nature of PTSD and its impact on individuals, families, and communities, especially in conflict-prone or conflict-affected societies.

## 1. Introduction

The American Psychiatric Association defines post-traumatic stress disorder (PTSD) as a trauma-and stress-related disorder that develops in distressed individuals owing to traumatic events such as natural disasters, accidents, death, crime (sexual or otherwise), or war (American Psychiatric Association, 2013); and includes behavioral and action-level disorders that impact daily life. Global data from 2022 indicates a 16% prevalence of PTSD worldwide, and from 1989 to 2019, the prevalence of PTSD in war-afflicted countries was 23% (Hoppen et al., 2021).

Approximately half of the world’s countries have been afflicted with war or an open conflict over the past three decades (Pettersson and Wallensteen, 2015). Most war casualties are civilians, with multitudes living in low-and middle-income countries that are perpetually riddled with poverty and civil conflicts (Brundtland, 2000). These environmental stressors contribute to mental disorder morbidity, including anxiety, depression, and PTSD (Yatham et al., 2018). The lack of competent mental health personnel—owing to insufficient mental health services—poses challenges in the implementation of evidence-based interventions customized to the needs of low middle-income countries (LMICs; Morina et al., 2010a). War-afflicted communities display a significantly higher prevalence of PTSD and depression than those with no recent history of open conflict (Priebe et al., 2010).

In this context, exploring the mental health status in Kosovo is critical. The Kosovo War ended in the 1990s, and the country witnessed multiple traumatic events during that time. Approximately 25–30 people per 100,000 population died, most of whom were Kosovar Albanians (Fanaj et al., 2014). In 2006, PTSD had a 22% prevalence rate in Kosovo, which reduced to 17.6% by 2013 (Fanaj and Melonashi, 2017); however, it was still substantially high, considering the country’s small population.

Kosovo, officially the Republic of Kosovo, is located in southeastern Europe. Albanians (67.1%) and Serbs (23.5%) were identified as the predominant ethnic groups in Kosovo by the 1961 population census (Statistical Office of Kosovo (SOK), 2008). Interethnic tensions between Albanians and Serbs were recurring and worsened throughout the late 1900s, culminating in the Kosovo War, which began in February 1998 and lasted until June 1999 (Malcolm, 1998). Over a million ethnic Albanians were forcefully driven from Kosovo during the conflict. The remaining civilians were subjected to war crimes and were victims of extreme abuse through rape, torture, looting, pillaging, and extortion. Working in North Macedonia’s refugee camps in 1999, Theresa Agovino remarked that Albanians were “victims of the worst ethnic cleansing in Europe since World War II” (Agovino, 1999). On 11 June 1999, international intervention and the deployment of National Atlantic Treaty Organization forces brought the war to an end. Fanaj et al. (2014) estimated that more than 18,000 individuals were killed during the Kosovo War, with approximately two-thirds of the casualties being civilians and more than 3,900 individuals being reported missing. The 2021 population census indicated that 88% of the population in Kosovo was Albanian, 7% were Serbs, and 5% were other ethnic groups (Statistical Office of Kosovo (SOK), 2008).

The study of Danuza et al. (2014) showed that 47.3% of the 150 veterans in Kosovo manifested PTSD symptoms 10 years after the war and that in this population, high PTSD prevalence was associated with a lack of social support and a low socioeconomic status. Ramsey (2017) surveyed PTSD prevalence among Albanian women living in Kosovar communities as survivors of extensive trauma. He explored the effect of culture on the perception of the causes and context of trauma, the barriers to seeking support, and women’s coping strategies for PTSD. The research highlighted the importance of community support for coping with trauma. Women identified their prime coping behaviors as talking to their friends, having faith, and working hard; they also discussed the vital role of their husbands and families in helping them cope with trauma. A survey of Kosovar refugees in the US found that 60.5% of respondents had PTSD symptoms (Ai et al., 2002). Respondents with multiple experiences of traumatic events were more prone to experiencing PTSD, as were most women.

The long-term effects of war-related PTSD have also been studied; PTSD had a 33% prevalence rate in the community even 8 years after the Balkan wars (Eytan et al., 2015). A sizable epidemiological survey of the Balkans determined that, of 648 Kosovar adults, 47.6% suffered from mood disorders and 41.8% were affected by anxiety (Priebe et al., 2010). Morina et al. (2010b) examined the diagnostic concordance between prolonged grief disorder (PGD), major depressive disorder (MDD), and PTSD among the bereaved survivors of the Kosovo War. Of the total sample, 38.3% were diagnosed with PGD, 55.0% with PTSD, and 38.3% with MDD. Women were more likely to be afflicted with PGD, and men were more likely to seek help for expediting recovery. Perpetually hazardous conditions were associated with an elevated risk of PTSD and MDD, but not of PGD.

Research indicates that Kosovo has a lower access to mental health services than other countries (Duraku and Hoxha, 2022). Moreover, Kosovars tend to be less help-seeking, implying that the challenge of addressing mental health is high (Duraku, 2021). According to the study of Shahini and Shala (2016), 11.2% of veterans were affected by PTSD symptoms even 8 years after the war ended, and many of them (6%) did not seek professional help for mental health. The approach toward treating mental disorders is mainly medical, whereas counseling services are relatively new in the Kosovo system and are mainly offered by private practices. Mental health literacy among the Kosovar population remains low, and the stigma toward seeking psychological help adds to their challenges (Fetahu, 2022; Ministry of Health in Kosovo, 2022).

The high incidence of PTSD in the Kosovar population indicates that the trauma is war-induced and has other far-reaching consequences. The war’s memories affect individuals and society as a whole. With approximately one-sixth of the population living below the poverty level and one-third of the working population being unemployed, Kosovar civilians are prone to chronic depression (Morina et al., 2010a; Eytan and Gex-Fabry, 2012; Van der Veen et al., 2015).

These findings indicate that PTSD is a significant public health issue in Kosovo. However, existing data regarding PTSD prevalence, severity, and the status of war veterans in the context of their help-seeking behavior and the need for mental health services are severely fragmented. There is also a lack of data that assess intergenerational trauma among parents (who experienced the Kosovo War) and their children (born after the war; Priebe et al., 2010). Intergenerational trauma is often defined as the transfer of the effects of trauma from one generation to the next. Intergenerational transmission of trauma can occur through direct transmission (e.g., stories told by survivors), indirect transmission (e.g., family dynamics), and biological factors (e.g., epigenetic changes; Kellermann, 2001).

The lack of structured datasets impairs the ability of both public and private agencies to develop population-level mental health interventions and programs. The lack of official data on PTSD prevalence and the limited research on this topic make it challenging for mental health professionals to design customized intervention programs. Therefore, this study aimed to obtain insights into the prevalence of perceived PTSD symptoms and its relationship with the traumatic experiences of two generations, parents and children (particularly those in their youth), and to understand their intergenerational trauma. The secondary aim of this study was to evaluate what kind of social support system is currently available to the target population to address PTSD and its associated issues, while describing their need for mental health services.

## 2. Methods

### 2.1. Sampling techniques and participants

The present study conducted a cross-sectional, quantitative analysis of the Kosovar Albanian population.

This study employed stratified random sampling, a probability sampling method that categorizes a large population into non-overlapping, discrete, and smaller subgroups known as strata. The strata were based on all of Kosovo’s districts (regions), including Ferizaj, Gjakova, Gjilan, Mitrovica, Peja, Pristina, and Prizren. The subgroups were chosen using a simple random sampling method known as “random quota sampling” to ensure that each district’s population had an equal chance of being chosen. To estimate the minimum required sample size for the study, a G*power analysis was performed, considering several parameters such as the desired level of the statistical power, significance level, and effect size. The results of the G*power analysis indicated that a sample size of *N* = 237 was required to ensure that the study was appropriately powered to achieve reliable and meaningful results.

The questionnaires were distributed via the Google Forms survey platform. Before commencing the survey, all participants provided informed consent, which included information about the study’s objective, the voluntary nature of participation, and data confidentiality. The study had an 86% response rate, showing a high degree of participation and engagement from the participants. The questionnaire took approximately 25 min to complete. The data was collected from September 2021 to April 2022.

### 2.2. Participants

Of the total sample (*n* = 237), 51.1% (*n* = 121) were parents and 48.9% (*n* = 116) were youth. The average age of the parents was 49.40 years (SD = 6.20), while that of the youth was 20.43 years (SD = 14). Among the overall participants, 69.6% were women. Most of the participants (*n* = 175, 75%) had no experience with receiving mental health services (Table 1). From the cities of each of Kosovo’s seven districts, the following percentages of participants were selected: Ferizaj: 6.5%, Gjakova: 13.1%, Gjilan: 8.5%, Mitrovica (south) 11.7%, Peja: 4.5%, Pristina: 47.1%, and Prizren: 8.6%.

**Table 1:** Participants’ demographic characteristics.

	Sample	Mean	SD	Minimum	Maximum	%
Age (years)	237	35.22	15.25	18	71	
**Gender**						
Woman	165					69.6
Man	72					30.4
**Generations**						
Youth	116	20.43	2.14	18	32	48.9
Parent	121	49.40	6.20	39	71	51.1
**Level of education**						
High school	65					26.6
Pursuing undergraduate studies	104					42.6
Bachelor’s degree	30					12.3
Pursuing postgraduate studies	6					2.5
Master’s degree	20					8.2
Elementary school	12					4.9
**Financial conditions**						
Very low economic status/extreme poverty	1					0.4
Average family income	195					82
Above average economic status/ high income	33					14
Affluent/economically prosperous/financially stable family	8					3.4
**Marital status**						
Married	119					50.21
Single	94					40
Divorced	1					0.4
Widowed	2					0.8
In a relationship	20					8.43

### 2.3. Procedures and measures

The variables in the study were assessed using the measures provided in this section. To assure accuracy, the questionnaires were translated into Albanian and then back into English. Prior to the main data collection, a pilot study was conducted to ensure the validity of the questionnaires. The pilot study involved a (*n* = 25) participants who completed the questionnaires and provided feedback on the items’ clarity, relevance, and comprehensibility. Adjustments were made based on their feedback to improve the validity and reliability of the measures employed in this study.

#### 2.3.1. PTSD checklist with life events checklist and criterion A

The PTSD Checklist for DSM-5 (PCL-5) is a 20-item self-report questionnaire that is designed according to the criteria set by the *Diagnostic and Statistical Manual of Mental Disorders Fifth Edition* (DSM-5; Weathers et al., 2013). It is used to screen individuals for PTSD, monitor symptom changes over the preceding month, and make a probable diagnosis of PTSD (Weathers et al., 2013). The questionnaire is scored using a five-point Likert scale (from 0 = no effect and 4 = extreme stress). Diagnosis using PCL-5 can be provided in two ways: by summing all 20 items (range 0–80) and using a cut-point score of 31–33. The PCL-5 is a well-known measure used in PTSD research and has demonstrated excellent internal consistency (α = 0.94; Blevins et al., 2015). Similar results were shown for the current study sample (α = 0.96). The Life Events Checklist for the DSM-5 (LEC-5) is another section of the questionnaire and is usually used in combination with the PCL-5. The LEC-5 is a self-report measure designed to screen for potentially traumatic events a person experienced during their lifetime that can cause distress, or potentially, PTSD. Items are scored on a six-point nominal scale (1 = It did not happen, 2 = Not sure, 3 = Part of my job, 4 = Learned about it, 5 = Witnessed it, and 6 = Happened to me; Weathers et al., 2018).

#### 2.3.2. Multidimensional scale of perceived social support

The Multidimensional Scale of Perceived Social Support (MSPSS), developed by Zimet et al. (1988) measures perceived social support. Three crucial aspects: family, friends, and significant others, are embedded in this 12-item questionnaire; a seven-point Likert-type scale (from 1 = very strongly disagree to 7 = very strongly agree) is used to rate each item. As age groups and cultural differences heavily influence social support, the validity and reliability of the MSPSS need to be substantiated in various populations (Dambi et al., 2018). A high score indicates high perceived social support, whereas a low score indicates scarcity, deprivation, or an absence of perceived social support (Eker et al., 2001). The scale’s reliability was evaluated using the Cronbach’s alpha coefficient, which was calculated by summing the results of all items. A score of 0.960 ensured the scale’s high internal consistency.

#### 2.3.3. Demographics and available support services

The questionnaire also contained questions collecting participants’ socio-demographics (e.g., age, gender, marital status, income) and information about resources available in case participants needed support owing to the nature of the questions asked.

### 2.4. Data analysis

The data were carefully examined using standard testing procedures; no missing or invalid data were detected. The normality of the data, presence of extreme outliers, and the size of the comparison groups met the required criteria for nonparametric analysis.

Descriptive statistics were employed to assess the demographic characteristics of the study sample, including age, gender, generation, education, financial conditions, marital status, and experience with mental health services.

To investigate the relationship between perceived social support and PTSD, a Spearman’s correlation test was conducted.

Furthermore, the Mann–Whitney *U-*test was utilized to compare the perceived social support and experiences of trauma and mood fluctuations between two independent groups, namely youth and parents.

The subsequent analyses, including the Spearman’s correlation and Mann–Whitney *U-*tests, consistently supported the nonparametric results. Additionally, both nonparametric and parametric analyses were conducted concurrently to ensure robustness of the findings and to address potential issues related to the normality of variable distribution (such as skewness or kurtosis).

## 3. Results

Among the participants, 78 (32.9%) met the criteria for PTSD (PCL-5 score > 31). Table 2 displays the PTSD levels of participants based on their socio-demographic attributes. For each category, the number of individuals with PTSD symptoms (scores>31) and those without PTSD symptoms (scores < 30) were calculated.

**Table 2:** Cut-off scores of perceived PTSD based on socio-demographic characteristics.

	Level of perceived PTSD	
	Lower than 30	31–33 or higher
**Gender**		
Man	52 (22%)	20 (8.4%)
Woman	107 (45.1%)	58 (24.4%)
**Generation**		
Youth	65 (27.4%)	51 (21.5%)
Parents	94 (40%)	27 (11.4%)
**Level of education**		
High school	49 (20.7%)	16 (6.8%)
Pursuing undergraduate studies	58 (24.5%)	46 (19.4%)
Bachelor’s degree	26 (11%)	4 (1.7%)
Pursuing postgraduate studies	4 (1.7%)	2 (0.9%)
Master’s degree	13 (5.5%)	7 (3%)
Elementary school	9 (3.8%)	3 (1.26%)
**Financial conditions**		
Very low economic status/extreme poverty	1 (0.45%)	0 (%)
Average family income	131 (55.3)	64 (27%)
Above average economic status/high income	22 (9.3%)	11 (4.65%)
Affluent/economically prosperous/financially stable family	5 (2.1%)	3 (1.26%)
**Marital status**		
Married	93 (39.2)	26 (11%)
Single	58 (24.5)	36 (15.1%)
Divorced	1 (0.45)	0
Widowed	1 (0.45)	1 (0.45%)
In a relationship	4 (1.7%)	16 (6.75%)
**Previous experience with mental-health services**		
Yes	5 (2.15%)	53 (22.7%)
No	61 (26.1%)	114 (49%)

As shown in Table 3, perceived social support (significant other support, family support, and friends’ support) had a moderately negative and significant relationship with PTSD (Criterion B: Re-experiencing; Criterion C: Avoidance; Criterion D: Negative cognition and mood; and Criterion E: Arousal). This negative correlation indicates a positive effect of perceived social support in treating PTSD-afflicted individuals.

**Table 3:** Correlation between perceived social support and PTSD.

	Perceived PTSD	Re-experiencing	Avoidance	Negative cognition and mood	Arousal
Perceived social support	−0.376^**^	−0.275^**^	−0.355**	−0.348^**^	−0.414^**^
Significant other support	−0.266^**^	−0.189^**^	−0.265^**^	−0.272^**^	−0.272^**^
Family support	−0.371^**^	−0.291^**^	−0.305^**^	−0.359^**^	−0.386^**^
Friends support	−0.275^**^	−0.175^**^	−0.288^**^	−0.256^**^	−0.33^**^

Correlation is significant at the 0.01 level (2-tailed).

Table 4 presents descriptive data and results of the Mann–Whitney U test for generational differences in perceived social support and PTSD. The results show significant differences in PTSD between the youth and parents, with the parents scoring higher in terms of receiving perceived social support. This table corroborates the results of Table 3, which reported significantly higher levels of PTSD and lower levels of perceived support among youth than among parents (Criterion D: Negative cognition and mood, and Criterion E: Arousal).

**Table 4:** Comparison between groups regarding perceived social support and PTSD.

	Groups	*N*	Mean	Z	*P*
Perceived social support	Youth	116	99.35	−4.329	0.000
	Parent	121	137.84		
Significant other support	Youth	116	96.77	−4.998	0.000
	Parent	121	140.31		
Family support	Youth	116	97.96	−4.710	0.000
	Parent	121	139.17		
Friends support	Youth	116	113.47	−1.221	0.222
	Parent	121	124.30		
PPTSD	Youth	116	130.82	−2.602	0.009
	Parent	121	107.67		
Re-experiencing	Youth	116	124.55	−1.225	0.220
	Parent	121	113.68		
Avoidance	Youth	116	127.61	−1.924	0.054
	Parent	121	110.75		
Negative cognition and mood	Youth	116	133.96	−3.304	0.001
	Parent	121	104.66		
Arousal	Youth	116	131.70	−2.805	0.005
	Parent	121	106.82		

Correlation is significant at the 0.01 level (2-tailed).

Table 5 displays descriptive data as well as the results of the Mann–Whitney U test to assess for differences in PTSD prevalence and perceived social support between youth with parents with and without PTSD symptoms. Thus, the results show that youth with parents afflicted with PTSD were more prone to be afflicted themselves than were those whose parents did not have PTSD.

**Table 5:** Perceived PTSD and perceived social support among youth who have parents with PTSD and those who have parents that do not meet the criteria for PTSD.

	Groups	*N* ^*^	Mean	Z	*P*
Perceived social support	Parent with PTSD	14	58.21	0.034	0.973
	Parent without PTSD	102	58.54		
Significant other support	Parent with PTSD	14	57.86	0.077	0.939
	Parent without PTSD	102	58.59		
Family support	Parent with PTSD	14	50.46	0.959	0.337
	Parent without PTSD	102	59.6		
Friends support	Parent with PTSD	14	62.36	0.461	0.645
	Parent without PTSD	102	57.97		
PTSD	Parent with PTSD	14	82.43	2.842	0.004
	Parent without PTSD	102	55.22		
Re-experiencing	Parent with PTSD	14	80	−2.56	0.01
	Parent without PTSD	102	55.55		
Avoidance	Parent with PTSD	14	72.21	1.647	0.1
	Parent without PTSD	102	56.62		
Negative cognition and mood	Parent with PTSD	14	87.57	3.457	0.001
	Parent without PTSD	102	54.51		
Arousal	Parent with PTSD	14	77.32	2.239	0.025
	Parent without PTSD	102	55.92		

^*^N = 14 represents the number of youth whose parents meet the criteria for perceived PTSD based on the cut-off (scores > 31), whereas *N* = 102 represents the number of youth whose parents do not meet such criteria.

The results indicate no significant differences between boys/men and girls/women in terms of PTSD level (Z = −0.903 *p* > 0.05) or perceived social support (Z = −0.874, *p* > 0.05) among the overall participants. Furthermore, no significant gender differences in PTSD symptoms were noticed among youth whose parents had PTSD.

The results indicate significant differences in the traumatic experiences reported by the youth and parents. Parents reported more traumatic experiences owing to natural disasters (Z = −2.214, *p* = 0.027) and warfare (Z = −7.513, *p* < 0.001). Meanwhile, the youth reported more experiences of trauma induced by physical assault (Z = −2.727, *p* = 0.006), sexual assault (rape, attempted rape, and coerced sexual acts; Z = −3.809, *p* < 0.001), and other unwanted or uncomfortable sexual experiences (Z = −2.445, *p* = 0.015).

Regarding the comparison of traumatic experiences between youth whose parents did and did not have PTSD, the results indicate that youth with parents who had PTSD had significantly higher traumatic experiences, such as exposure to sudden violent death (Z = −1.969, *p* = 0.049) and accidental death (Z = −2.633, *p* = 0.008) and experiences of assault with a weapon (Z = −1.945, *p* = 0.052), sexual assault (Z = −1,474, *p* = 0.141), and captivity (Z = −1.17, *p* = 0.242), compared to youth with parents without PTSD (Table 6). Moreover, regarding gender-based differences in the exposure to stressful events for the overall sample, boys/men had significantly higher scores on combat or war zone exposure (Z = −5.116, *p* < 0.001), weapon assault (Z = −3.32, *p* = 0.001), physical assault (Z = −2.218, *p* = 0.027), and transportation accidents (Z = −3.007, *p* = 0.003) than girls/women did.

**Table 6:** Differences in the traumatic experiences of youth who have parents with and without PTSD.

	Groups	*N* ^*^	Mean	Z	*P*
Natural disaster (for example, flood, hurricane, tornado, earthquake)	Parent with PTSD	14	68.68	−1.301	0.193
	Parent without PTSD	102	57.10		
Fire or explosion	Parent with PTSD	14	64.11	−0.74	0.459
	Parent without PTSD	102	57.73		
Transportation accident (for example, car accident, boat accident, train wreck, plane crash)	Parent with PTSD	14	52	−0.816	0.415
	Parent without PTSD	102	59.39		
Serious accident at work, home, or during recreational activity	Parent with PTSD	14	59.29	−0.242	0.808
	Parent without PTSD	102	57.25		
Exposure to a toxic substance (for example, dangerous chemicals, radiation)	Parent with PTSD	14	56.89	−0.258	0.796
	Parent without PTSD	102	58.72		
Physical assault (for example, being attacked, hit, slapped, kicked, or beaten up)	Parent with PTSD	14	73.14	−1.821	0.069
	Parent without PTSD	102	56.49		
Assault with a weapon (for example, being shot, stabbed, or threatened with a knife, gun, or bomb)	Parent with PTSD	14	71.07	−1.945	0.052
	Parent without PTSD	102	56.19		
Sexual assault (rape, attempted rape, made to perform any type of sexual activity through force or threat of harm)	Parent with PTSD	14	68.14	−1.474	0.141
	Parent without PTSD	102	57.18		
Other unwanted or uncomfortable sexual experiences	Parent with PTSD	14	76.82	−2.44	0.015
	Parent without PTSD	102	54.8		
Combat or exposure to a warzone (in the military or as a civilian)	Parent with PTSD	14	54.8	−0.476	0.634
	Parent without PTSD	102	58.06		
Captivity (for example, being kidnapped, abducted, held hostage, or prisoner of war)	Parent with PTSD	14	65.11	−1.17	0.242
	Parent without PTSD	102	57.59		
Life-threatening illness or injury	Parent with PTSD	14	63.79	−0.789	0.43
	Parent without PTSD	102	57.2		
Severe human suffering	Parent with PTSD	14	65.14	−0.911	0.362
	Parent without PTSD	102	57.59		
Exposure to sudden violent death (for example, homicide, suicide)	Parent with PTSD	14	72.07	−1.969	0.049
	Parent without PTSD	102	56.64		
Exposure to sudden accidental death	Parent with PTSD	14	76.96	−2.633	0.008
	Parent without PTSD	102	55.97		
Serious injury, harm, or death you caused to someone else	Parent with PTSD	14	60.43	−0.348	0.728
	Parent without PTSD	102	58.24		
Any other very stressful event or experience	Parent with PTSD	14	71.04	−1.784	0.074
	Parent without PTSD	102	55.61		

Participants with perceived PTSD and lower perceived social support reported a significantly greater need for mental health services than those without PTSD symptoms did (Z = −3.453, *p* = 0.001). Lastly, participants who experienced traumatic events related to natural disaster (Z = −0.681, *p* = 0.496); serious accidents at work, home, or during recreational activity (Z = −2.798, *p* = 0.005); exposure to a toxic substance (Z = −3.552, *p* = 0.000); physical assault (Z = −2.906, *p* = 0.004); sexual assault (Z = −3.076, *p* = 0.002); life-threatening illness or injury (Z = −3.59, *p* = 0.000); severe human suffering (Z = −3.04, *p* = 0.002); exposure to sudden violent death (Z = −3.262, p = 0.001) or accidental death (Z = −2.225, *p* = 0.026); and any other very stressful event or experience (Z = −4.032, *p* = 0.000), reported a significantly higher need for mental health services compared to those who did not experience these events.

## 4. Discussion

This study found a significant prevalence of perceived PTSD and a lack of social support-seeking behavior in the target population. The study parameters included the different PTSD symptoms and traumatic experiences of the participants.

The finding that parents in the study scored higher in terms of perceived social support is consistent with those of previous research on the relationship between age and social support (Kafetsios and Sideridis, 2006; Mitchell et al., 2020). Parents tend to have larger social networks and may have developed stronger and more stable relationships over time, which can contribute to their perception of higher social support. The significant differences in perceived PTSD between youth and parents reported in this study are also consistent with previous research on the impact of trauma on different age groups (Finkelhor et al., 2007).

Our study’s result is consistent with those of existing studies, as a negative correlation with different social systems implies that an increase in support systems will reduce PTSD symptoms among individuals, accompanied by subjective well-being (Kelmendi and Hamby, 2022). The Mann–Whitney *U t*-test results revealed that perceived social support, family support, and other support systems significantly impact PTSD and the severity of the symptoms. The difference in support systems and their impact on different population groups highlight the role socio-economic factors can play in their access to social support (Kamberi and Abazi, 2021; Fel et al., 2022). Additionally, significant differences were observed between the youth and parents with respect to PTSD, negative mood and cognition, and arousal symptoms, consistent with other studies (Kelmendi et al., 2022; Kelmendi and Hamby, 2022).

As observed from this study’s results, youth whose parents had PTSD were more prone to experiencing PTSD symptoms than those whose parents did not have PTSD were. Although this result suggests that intergenerational trauma resulting from war-induced PTSD is prevalent among Kosovar Albanians, the lack of social support, mental health literacy, including social stigmas associated with mental disorders and toward those seeking psychological help, as well as a limited access to mental health services may have worsened the mental wellbeing and trauma-related symptoms among populations at risk for mental health issues (Eker et al., 2001; Ministry of Health in Kosovo, 2022). Similar factors, such as poverty, lack of education, cultural stigma, and limited access to services were also among the main barriers to seeking help for mental health issues among war-affected individuals in previous studies conducted in Kosovo (Kamberi and Abazi, 2021).

### 4.1. Implications for practice

A fundamental principle of global mental health is that interventions must be both practical and scalable for enabling rapid implementation, even in LMICs, regardless of these nations’ limited resources (Tol et al., 2011). According to research conducted in Western countries, psychological therapies can effectively treat PTSD and its accompanying symptoms (Bisson et al., 2013; Ehring et al., 2014). As noted in the current study’s results, the participants reported a higher need for mental health services. However, only a few of them benefited from such services. Therefore, it is necessary to improve the accessibility for psychological support while increasing public awareness about the importance of receiving professional support and decreasing stigma toward those seeking help.

Furthermore, mental health interventions should lay the foundation for long-term psychosocial support programs and ensure institutional development (Abramowitz, 2010). Augmenting government policies, finances, research, and human resources (training along with designing programs and services) should be the primary focus of these interventions (Ventevogel et al., 2011).

Specialized cognitive behavioral therapy treatments that target trauma have produced significantly positive results (Schick et al., 2013). The involvement of trauma survivors in mental health teams should also be considered (Van Ginneken et al., 2013). Many European countries are reported to have taken important steps toward better provision of care for trauma survivors. The European Society of Traumatic Stress Studies, which is an umbrella organization for European societies, consists of members from numerous European countries (i.e., Austria, Belgium, Croatia, Georgia, Lithuania, Italy, The Netherlands, Poland, Portugal, Sweden, the German and French-speaking parts of Switzerland, Ukraine, and the United Kingdom). Therefore, Kosovo should consider the possibility of joining such societies, learning through others about practices that can advance evidence-based practices, and strengthening cooperation to advance the support for its citizens’ mental health and trauma-related issues (Schäfer et al., 2018).

Clinicians should explore support prospects through a combination of interventions and counseling tailored to the client’s trauma and cultural background for optimal remedial treatment. For instance, recent research suggests that mental-health-service-utilization rates in Kosovo are ineffective in improving the mental health of the population (Morina et al., 2010a; Duraku, 2021). Therefore, more studies should be conducted to evaluate the quality of mental healthcare services, especially to identify needs and barriers. Poverty and economic crises are evident in people’s accounts of economic hardships and the barriers they create in accessing professional help in Kosovo (Ramsey, 2017). Several other studies have recognized that professional services are not available to most of the population in Kosovo (Eytan et al., 2015). However, there is a compelling demand for these services (Van der Veen et al., 2015).

This study’s results highlight the importance of considering age as a factor for assessing and treating PTSD. Mental health professionals should recognize the unique needs and experiences of different age groups and tailor interventions accordingly. For example, interventions for parents may focus more on maintaining social support networks, whereas those for the youth may involve strengthening social support, coping strategies, and resilience. Moreover, while evaluating the efficacy of the currently available treatments, considering the advancement of mental health providers’ capacities through professional development on new intervention alternatives, such as psychosocial interventions for trauma, is crucial. Adapting educational policies and helping schools and the staff to create trauma-informed environments could also support the advancement of help-seeking behaviors following a trauma (Blodgett and Dorado, 2016; Chafouleas et al., 2016).

In addition to having the implications for mental health practice and policy, the study’s findings have broader implications for addressing the impact of war and conflict at individual and societal levels. The high prevalence of perceived PTSD symptoms among parents and the youth in Kosovo underscores the long-lasting and profound impact of war-induced trauma. Furthermore, this study highlights the need for comprehensive interventions that address the intergenerational nature of PTSD and promote healing and resilience as well as a multidisciplinary approach to understand the impact of war-induced trauma, involving not only mental health professionals but also educators, community leaders, and policymakers. Such an approach could promote greater understanding of the impact of war on individuals and communities, and foster more effective interventions to address its effects (Ciaramella et al., 2022).

### 4.2. Limitations and future research directions

Although we addressed the ethnocultural factors and intergenerational effects of perceived PTSD among the youth born after the Kosovo war and parents who experienced war, more conclusive studies are imperative to explore the increasing need for customized therapy for specific individuals or groups.

The study has several limitations that should be considered while interpreting its findings. The sample size was limited, particularly for the group of youth whose parents had experienced PTSD. This might have limited the generalizability of the findings to other populations, and the current study findings should thus be interpreted with caution. This study relies on self-reported measures, which may be biased or inaccurate. The current study had few male participants who satisfied the PTSD criteria, which influenced the comparative analysis for gender differences in PTSD symptoms and perceived social support. Finally, despite the fact that the two groups (parents/youth) were matched, they were not divided equally. In a few cases, both parents of the same youth participated in the study. Another limitation of this study is that it may not have effectively reflected the complexity of ethnic diversity among the Kosovo war-affected community. Future research is required to better understand the intergenerational impacts of PTSD in people of diverse ethnic backgrounds in Kosovo. In post-conflict settings, ethnicity shapes an individual’s experiences, coping mechanisms, and social support networks. Cultural values, traditions, and historical context can all influence how individuals interpret and respond to traumatic events. According to research, various ethnic groups can have varied post-conflict experiences and responses to trauma (Miller and Rasmussen, 2010).

Future research should overcome these limitations by conducting larger-scale studies in diverse cultural contexts and adopting a variety of assessment methods. Additionally, it should investigate the impact of trauma on other outcomes, such as physical health, academic achievement, and social functioning. Future studies may also include resilience and vulnerability factors, sociocultural factors, training needs of professionals, and other barriers to care.

Additionally, scholars should conduct longitudinal studies involving multivariate analyses with larger sample sizes to investigate these indicators as well as causal and resilience factors. Longitudinal studies may help investigate the long-term impact of trauma and identify potential protective factors that may promote resilience in individuals and communities affected by war-induced trauma.

## Data availability statement

The raw data supporting the conclusions of this article will be made available by the corresponding author upon reasonable request.

## Ethics statement

The study was approved by the University of Prishtina, Faculty of Philosophy. The participants provided their written informed consent to participate in the study.

## Author contributions

ZH developed the research idea and contributed to all parts of the manuscript development and finalization. ZH, GJ, and DG have been involved in data gathering, analysis, literature review, and manuscript development from the initial phases to the finalization of the manuscript. All authors contributed to the article and approved the submitted version.

## Conflict of interest

The authors declare that the research was conducted in the absence of any commercial or financial relationships that could be construed as a potential conflict of interest.

## Publisher’s note

All claims expressed in this article are solely those of the authors and do not necessarily represent those of their affiliated organizations, or those of the publisher, the editors and the reviewers. Any product that may be evaluated in this article, or claim that may be made by its manufacturer, is not guaranteed or endorsed by the publisher.
